# Decentralised paediatric HIV care in Ethiopia: a comparison between outcomes of patients managed in health centres and in a hospital clinic

**DOI:** 10.3402/gha.v6i0.22274

**Published:** 2013-11-11

**Authors:** Oskar Hagströmer, Lars Lundstedt, Taye Tolera Balcha, Per Björkman

**Affiliations:** 1 Infectious Disease Research Unit, Department of Clinical Sciences, Malmö, Lund University, Malmö, Sweden; 2 Oromia Regional Health Bureau, Addis Abeba, Ethiopia; 3 Federal Ministry of Health, Addis Abeba, Ethiopia

**Keywords:** primary health care, paediatric, HIV, Ethiopia, ART, decentralisation

## Abstract

**Background:**

In order to increase access to antiretroviral therapy (ART) in HIV-infected children, paediatric HIV care has been introduced in health centres in Ethiopia, where patients are managed by health professionals with limited training.

**Objective:**

To compare outcomes of paediatric HIV care in hospital and health centre clinics and to determine risk factors for death and loss to follow-up (LTFU).

**Design:**

Retrospective comparison of patient characteristics and outcomes among children managed in a public hospital and all five public health centres in the uptake area.

**Results:**

Among 1,960 patients (health centres 572, hospital clinic 1,388), 34% were lost to follow-up, 2% died, 14% were transferred out, and 46% remained in care. Children initiating ART in the hospital clinic had lower median CD4 cell counts (age <1 year: 575 vs. 1,183 cells/mm^3^, *p*=0.024; age 1–5 years: 370 vs. 598 cells/mm^3^, *p*<0.001; age >5 years: 186 vs. 259 cells/mm^3^, *p*<0.001), and a higher proportion were <1 year of age (22% vs. 15%, *p*=0.025). ART initiation rates and retention in care were similar between children managed in health centres and in the hospital clinic (36% vs. 37% and 47% vs. 46%, respectively). Among patients starting ART, mortality was associated with age <1 year [hazard ratio (HR) 12.0; 95% confidence interval (CI): 3.5, 41]. LTFU was associated with CD4 cell counts <350 cells/mm^3^ (HR 1.8; 95% CI: 1.2, 3.0), weight-for-age *z*-scores below −4 (HR 2.8; 95% CI: 1.4, 5.6), and age <5 years (1–5 years: HR 1.6; 95% CI: 1.0, 2.5; <1 year: HR 3.3; 95% CI: 1.6, 6.6).

**Conclusions:**

Outcomes of HIV care were similar for Ethiopian children managed in a hospital clinic or in health centres. However, patients treated at the hospital clinic had characteristics of more advanced disease. Rates of LTFU were high in both types of health facility.

At the end of 2011, the number of HIV-infected children globally was estimated to be 3.4 million, 90% of whom lived in sub-Saharan Africa (1). Among these, the dominant route of HIV acquisition was through mother-to-child transmission (2, 3). Perinatally infected children have rapid disease progression; a majority will die before 2 years of age if antiretroviral therapy (ART) is not provided (4).

The proportion of HIV-infected individuals receiving ART has increased dramatically during the last decade, especially since 2009 (1). However, in 2010 only 23% of HIV-infected children received treatment and 250,000 died of HIV/AIDS (5). In order to reach the goal of universal access to ART in resource-limited settings, it is necessary to integrate this treatment within the framework of the primary healthcare system (6). Transfer of HIV care, including provision of ART, from doctors to nurses has been associated with excellent outcomes in adult patients in South Africa (7). In a recent study based on aggregate programme data from five sub-Saharan countries, lower rates of loss to follow-up (LTFU) and death were observed among children receiving ART in primary health facilities than in secondary health facilities (8). Additionally, satisfactory outcomes of paediatric HIV care in Zambian health centres have been reported (9).

Providing ART to children is more complex and challenging than treating adults. HIV-positive children are frequently confronted with a range of medical and psychosocial problems. Many such individuals are orphans, some of whom lack regular alternative caretakers (10). Concomitant health problems, such as malnutrition (11) and tuberculosis (TB), are common (12). Drug adherence, which is of critical importance for treatment outcome, may be more problematic in children than in adults (4). Furthermore, the dosing of antiretroviral drugs in children has to be continuously modified during growth.

In Ethiopia, decentralisation of both adult and paediatric HIV care was begun in 2005, and antiretroviral drugs are provided free of charge through a national programme. The number of HIV-infected Ethiopian children was estimated to be 80,000 in 2010 (13); however, according to the 2012 UNAIDS report, only one in five eligible children receive ART (1). A study conducted in Addis Abeba showed that the median age at ART initiation was 5.9 years and 6.3% died during their first year of ART (14). The decentralisation of HIV care calls for assessment of the outcome of care provided in health centres. Whereas hospitals provide the highest level of health facilities with a full cadre of health professionals available, medical staffing in health centres is restricted to nurses and health officers (with 4 years of academic training). In a study based on register data in the Oromia region from 2010, similar ART outcomes were found for adults treated in health centres and in hospital clinics (15). However, no such comparison has been performed for children, and published data on paediatric ART in Ethiopia have hitherto been restricted to hospital settings (14, 16, 17).

The main objective of this study was to compare the outcomes in HIV-infected children receiving care in health centres to those treated in a large hospital clinic in Ethiopia. In addition, we analysed risk factors for adverse treatment outcomes (death and LTFU) among HIV-infected children initiating ART in these health facilities.

## Methods

### Study setting

This study was conducted in Adama, the economic capital of the Oromia region in Ethiopia. Adama has around 300,000 inhabitants and is located on a major transport highway linking Addis Abeba and Djibouti. All public health facilities within a radius of 25 km providing HIV care were included as study sites: the Adama Regional Hospital [which had the largest number of patients on ART in Ethiopia in 2010 (18)] and the health centres of Adama, Geda, Wolenchiti, and Dera. Characteristics for the study sites are presented in Table 1.

**Table 1:** Characteristics of the study health facilities (respective numbers of all adult and paediatric patients registered for HIV care from initiation of the HIV programme to October 2012)

	Health facilities					
	
		Urban			Rural	
			
	Adama Hospital	Adama HC	Geda HC	Mojo HC	Dhera HC	Wolenchiti HC
Time initiation of ART programme	March 2005	October 2006	August 2007	September 2006	December 2006	March 2006
Total no. of pre-ART subjects registered	19,038	2,403	1,326	3,449	819	1,217
Total no. of ART recipients registered	11,233	1,058	641	1,576	448	579
Physicians	45	0	0	0	0	0
Health officers	7	4	3	4	1	4
Nurses	117	13	9	9	9	9

The number of staff refers to the whole facility. HC, health centre.

In the hospital clinic, children are managed by nurses specialised in paediatric HIV care, and physicians are available for consultation. In the health centres, nurses or health officers who have received basic training in the management of HIV infection and ART, manage patients. Physicians are not available in these facilities.

Irrespective of type of facility, all professionals involved in the management of HIV-infected patients are required to have completed formal training on the principles of ART (19).

All health facilities follow national guidelines for the management of HIV infection, and have access to similar drugs (both for ART and for the treatment of opportunistic infections) (2), as well as nutritional support. Both clinic visits and antiretroviral drugs are provided free of charge.

HIV diagnosis is based on rapid antibody tests according to national guidelines; for children below 18 months of age, detection of HIV DNA from dried blood spots or p24 antigen is used (20). All HIV-infected children are followed at monthly visits, before and after initiation of ART.

### Study design

All children (age below 15 years) with confirmed HIV infection registered in the study sites between 1 January 2007 and 1 July 2012, were included. No other exclusion criteria were applied. Before initiation of ART, subjects are registered as ‘pre-ART patients’, while all subjects who have started ART are henceforth categorised as ‘ART patients’.

Patients were included in the pre-ART cohort if ART was not prescribed within the first 30 days and in the ART cohort after treatment initiation. All were followed from the date of diagnosis until one of these outcomes: death, documented transfer to another health facility (transfer out), LTFU (defined as no registered clinic visit for more than 90 days since the last visit), start of ART (for patients not receiving ART within the first 30 days – the pre-ART cohort) or the end of study follow-up (1 July 2012).

Since this study was performed retrospectively and covered a time period of more than 5 years, tracing of patients registered as lost to follow-up was not possible.

For all included individuals, collected data comprised gender, age, and date at enrolment, date of last recorded clinic visit, type of health facility, type of primary caretaker and prescribed treatment for active TB. The number of days since the last clinic visit (from 1 July 2012) was calculated and used to determine whether a patient had been lost to follow-up. For patients on ART, the date at the start of ART (treatment start, TS) was also collected and used to calculate age at TS, number of days from diagnosis to TS and number of days on ART. For patients with missing date of birth (18.5% of the study population), reported age at the date of registration was used to estimate age.

Additionally, data on weight, WHO clinical stage, CD4 absolute cell count, and CD4 cell percentage were collected at three time points (TS, 5–7 months, and 11–13 months) after TS for patients on ART. For pre-ART patients, these data were only collected at their last visit.

### Data collection procedure

At Adama Regional Hospital, patients eligible for this study were identified from an existing database at this clinic. After each clinic visit, trained data clerks enter patient data into this database from patient cards. This database was also used for retrieving participant data for this study. To confirm the validity of the database, 173 out of 1,388 (12.5%) patient cards were collected and crosschecked with the database. The error rate was below 3%.

The health centres had no computerised databases; instead, study data were retrieved from patient registers and individual patient cards. At each site, patients matching inclusion criteria were identified using pre-ART and ART registers covering the study time periods. Medical record numbers were retrieved from these registers and used to find each individual patient card. The registers were also used to crosscheck pre-ART patients starting ART. In cases where individual patient cards could not be found (71/572, 12.4%), data on age at enrolment, gender, and study outcomes (in such cases the outcome were explicitly noted in the register as ‘lost’, ‘dead’ or ‘transfer out’) was entered from registers. Since visits at the health centres were not consistently entered in registers, this variable could not be studied for patients without patient cards. As no outcome was noted in the registers for 57/572 (10%) subjects, outcomes for these patients could not be assessed.

### Study sample

During the study period, 1,960 HIV-positive children were registered in the study health facilities. Among these, 1,388 were registered in the hospital clinic and 572 in the health centres (yielding 2,942 and 792 person-years of follow-up, respectively). During the study period, 1,537 children were registered as pre-ART patients, and at the end of the study period, 988 children were registered as having started ART. Fourteen patients from the pre-ART group who had started ART during follow-up were excluded from the ART group (age >15 years at TS for 11, register mismatches for three). Hence, data from 974 ART patients could be analysed.

### Statistical analysis

All data were entered into a Microsoft Excel version 14.0 spreadsheet and statistical analysis was performed with IBM SPSS statistical software version 20.0 using Mac OSX.

Patient characteristics were analysed using median and percentiles since the population was not normally distributed. Differences in groups between categorical variables were analysed using the χ^2^ test or Fisher's exact test, while differences between the two groups in continuous variables were analysed with the Mann–Whitney test. For analysis of CD4 absolute cell counts and percentages, the study cohort was divided into three groups: infants (0–1 years), toddlers (1–5 years), and older children (>5 years).

Adverse outcomes (LTFU and death) and retention in care were analysed using Kaplan–Meier plots, and statistical significance was calculated with the log-rank test. Death and LTFU were grouped together as adverse outcomes for this analysis due to a low proportion of reported deaths and a high proportion of LTFU.

Weights were plotted against US Centres for Disease Control and Prevention (CDC) charts (21) using Epi Info 7 (22) to calculate weight-for-age *z*-scores (WAZ). CDC charts were used in preference to WHO growth charts from 2007 (23) since they covered the ages of the study population (in contrast to the WHO charts).

Risk factors for death and LTFU were analysed separately, using crude and adjusted Cox proportional hazard ratios (HRs). Since an unknown proportion of patients lost to follow-up could have died, subjects with LTFU as a registered outcome were excluded from the analysis of risk factors for mortality, and likewise those with death as a registered outcome were excluded from the analysis of risk factors for LTFU. To ensure that the use of HRs was appropriate, the data were first tested for proportionality with Kaplan–Meier analysis. Since proportionality was only found for the first 3 years of follow-up, risk factors for adverse outcomes were only analysed for the first 1,000 days of ART.

For all statistical analyses, *p*-values below 0.05 were considered to be significant.

### Ethical considerations

Ethical approval was received from the Institutional Review Board of the Oromia Regional Health Bureau. Due to the retrospective design of this study, informed consent from participant patients was not obtained. To ensure patient confidentiality, all patient data were managed strictly under code, which was only accessible to the study investigators.

## Results

### Baseline characteristics

The baseline characteristics of participants with regard to type of health facility are presented in Table 2. The age distribution between the sites showed variations, which were most pronounced for infants and toddlers, with a greater proportion of infants in the hospital clinic and a higher proportion of toddlers in the health centres. Older children were evenly distributed.

**Table 2:** Characteristics of children receiving care in the hospital clinic and the five health centres

		Not initiating ART during follow-up			Initiating ART during follow-up		
			
		Health centre clinics	Hospital clinic	*p*	Health centre clinics	Hospital clinic	*p*
Female (%)		146 (49.0)	311 (46.1)	0.220^a^	144 (54.5)	349 (49.5)	0.092^a^
Male (%)		152 (51.0)	364 (53.9)		120 (45.5)	356 (50.5)	
Age at diagnosis median (5–95th percentile)		5 (1–12)	5 (0–13)	0.083^b^	5 (0–13)	5 (0–13)	0.349^b^
	Missing	0	0		9	0	
	0–1	17 (5.7)	123 (18.2)		40 (15.4)	158 (22.4)	
Age in years, count (%)	>1–5	143 (47.7)	256 (37.9)	<0.001^c^	94 (36.2)	207 (29.4)	**0.025** c
	>5–15	140 (46.7)	296 (43.9)		126 (48.5)	340 (48.2)	
Without parents (%)		37 (17.7)	197 (29.2)	<0.001^a^	56 (22.6)	227 (33.5)	**0.001** a
	Adjusted	−1.8 (−4.5 to 0.10)	−1.8 (−4.5 to 0.10)	0.798^b^	−2.5 − −6.8–0.08)	−2.5 (−6.2– − 0.3)	0.280^b^
Weight–for-age *z*-score median (5–95th percentiles)	Missing	115	187		39	47	
	Incl. outliers	−1.8 (−5.1 to 0.3)	−1.8 (−5.2 to 0.3)	0.800^b^	−2.6 (−9.9 to 0.1)	−2.5 (−6.6 to 0.3)	**0.045** b
	Missing	105	163		30	25	
	1 and 2	157 (77.7)	429 (82.7)		122 (46.7)	302 (44.1)	
WHO clinical stage (%)	3	41 (20.3)	82 (15.8)	0.312^c^	121 (46.4)	325 (47.4)	0.630^c^
	4	4 (2.0)	8(1.5)		18 (6.9)	58 (8.5)	
CD4 cell count at TS (cells/mm^3^) for different age groups median (5–95th percentile)	0–1	1,150 (598–1,701)	951 (45–2,000)	0.570^b^	1,183 (721–2,693)	575 (30–1,346)	**0.024** b
	>1–5	824 (264–2,020)	678 (214–1,637)	0.009^b^	598 (168–1,449)	370 (62–1,061)	**<0.001** b
	>5–15	548 (166–1,002)	485 (177–1,059)	0.098^b^	259 (71–861)	186 (28–543)	**<0.001** b
	Missing	144	208		52	106	
CD4% at TS for different age groups^d^ median (5–95th percentile)	0–1 years	22 (18–26)	25 (13–31)	0.658^b^	16.0 (12.0–37.9)	12.0 (3.0–40.0)	0.328^b^
	>1–5 years	21 (9–33)	20 (11–49)	0.719^b^	15.0 (6.0–23.0)	11.0 (4.0–38.0)	**0.032** b
	>5–15 years	20 (7–34)	22 (9–36)	0.997^b^	13.0 (5.0–36.0)	8.5 (2.0–20.0)	**0.001** b
	Missing	152	624		121	606	
TB treatment since diagnosis (%)		4 (1.7)	25 (3.7)	0.086^a^	12 (4.7)	109 (15.5)	**<0.001** a

ART, antiretroviral therapy; HC, health centre.

Data refer to the last recorded visit (for patients not initiating antiretroviral therapy (ART) during follow-up) and at date at start of ART [treatment start (TS) for patients initiating ART].

a Fisher′s exact test

b Mann–Whitney test

c χ^2^ test

d for patients not on ART, CD4 was measured at last visit; for patients on ART, CD4 was measured at date at start of ART (TS).

Significant p-values are marked with bold characters.

Overall, health centre patients had higher median CD4 cell counts than hospital patients. This was observed for all age groups, with statistical significance observed for patients starting ART. The same trend was noted for CD4 cell percentage, although this information was missing for a majority of patients. No significant difference in WAZ scores was found between patients treated in health centres and those treated in the hospital clinic [for this analysis, 31 patients with outlier values were excluded (18 with WAZ < − 10, and 13 with WAZ >4)]. No significant difference between the groups regarding WHO clinical stage was detected, although pre-ART patients in the hospital tended to be in stage 1–2 more frequently, with a higher proportion of stage 3 pre-ART patients in health centres. Patients receiving ART in the hospital were prescribed TB treatment to a higher extent (*p*<0.1), whereas there was no difference in rates of TB treatment between the sites for pre-ART patients. A higher proportion of health centre patients had parents as registered caretakers.

### Patient outcomes by type of health facility

At the end of follow-up, 612 (63%) ART and 299 (19%) pre-ART patients in the total study population remained in care. At that time point, 565 (37%) pre-ART patients had initiated ART (Table 3).

**Table 3:** Outcomes of patients with regard to type of health facility

	Health facility no. (%)							
	
				Urban health centres			Rural health centres	
	Total	Hospital clinic	Health centre clinics					
				Adama	Geda	Mojo	Dera	Wolenchiti
Total number of HIV-infected patients	1,960 (100)	1,388 (100)	572 (100)	107 (100)	77 (100)	235 (100)	90 (100)	63 (100)
Remaining in care	911 (46)	643 (46)	268 (47)	54 (50)	42 (55)	94 (40)	49 (54)	29 (46)
Dead	37 (2)	19 (1)	18 (3)	2 (2)	4 (5)	6 (3)	5 (6)	1 (2)
Lost to follow-up	664 (34)	493 (36)	171 (30)	28 (26)	23 (30)	81 (34)	20 (22)	19 (30)
Transferred out	277 (14)	225 (16)	52 (9)	12 (11)	5 (6)	22 (9)	5 (6)	8 (13)
Not assessable	57 (3)	0 (0)	57 (10)	9 (8)	2 (3)	31 (13)	10 (11)	5 (8)
Excluded	14 (1)	8 (1)	6 (1)	2 (2)	1 (1)	1 (0)	1 (1)	1 (2)
Patients on ART	974 (50)	705 (51)	269 (47)	44 (41)	44 (57)	106 (45)	43 (48)	32 (51)
Remaining in care	612 (63)	426 (60)	186 (69)	31 (70)	31 (70)	64 (60)	38 (88)	22 (69)
Dead	30 (3)	17 (2)	13 (5)	1 (2)	4 (9)	6 (6)	2 (5)	0 (0)
Lost to follow-up	178 (18)	134 (19)	44 (16)	5 (11)	7 (16)	23 (22)	2 (5)	7 (22)
Transferred out	148 (15)	128 (18)	20 (7)	7 (16)	2 (5)	8 (8)	1 (2)	2 (6)
Not assessable	6 (1)	0 (0)	6 (2)	0 (0)	0 (0)	5 (5)	0 (0)	1 (3)
Excluded	14	8	6	2	1	1	1	1
Patients initially in pre-ART care	1,537 (78)	1,076 (78)	461 (81)	86 (80)	61 (79)	190 (81)	73 (81)	51 (81)
Remaining in pre-ART care	299 (19)	217 (20)	82 (18)	23 (27)	11 (18)	30 (16)	11 (15)	7 (14)
Dead	7 (0)	2 (0)	5 (1)	1 (1)	0 (0)	0 (0)	3 (4)	1 (2)
Lost to follow-up	486 (32)	359 (33)	127 (28)	23 (27)	16 (26)	58 (31)	18 (25)	12 (24)
Transferred out	129 (8)	97 (9)	32 (7)	5 (6)	3 (5)	14 (7)	4 (5)	6 (12)
Initiating ART during follow-up	565 (37)	401 (37)	164 (36)	25 (29)	29 (48)	62 (33)	27 (37)	21 (41)
Not assessable	51 (3)	0 (0)	51 (11)	9 (10)	2 (3)	26 (14)	10 (14)	4 (8)

Results are separated depending on whether antiretroviral therapy (ART) was initiated during follow-up or whether the patient was initially in pre-ART care.

The proportions of patients starting ART during follow-up were similar in the hospital clinic and the health centres (51% vs. 47%; *p*=0.13). Some variations in ART initiation rates were observed among the different health centres (Table 3). Median follow-up time for patients on ART was longer in the hospital [3.1 years; interquartile range (IQR): 1.2–4.4 than in the health centres (2.2 years; IQR: 1.2–3.4), and the median time from diagnosis to TS was longer in the health centres (73 days; IQR: 19–298) than in the hospital (40 days; IQR: 10–263)].

Adverse outcomes (death or LTFU) were registered for 701 (36%) patients. Among 1,388 patients in hospital care, 512 (37%) had adverse outcomes, including 19 registered deaths (1%). In the health centres, 189 of 572 patients (33%) had adverse outcomes, with 18 deaths (3%; Table 3).

In the health centres, 15 participants died within 12 months after registration, generating a first-year mortality rate of 2.6% compared to 1.2% (16 patients) in the hospital. A total of 664 (34%) subjects were lost to follow-up, with a greater proportion in the hospital clinic (36% vs. 30% at the health centres; *p*=0.018). During the first year in care, 395 (28%) patients were lost from the hospital clinic compared to 132 (23%) patients in the health centres (*p*=0.008).

**Fig. 1:**
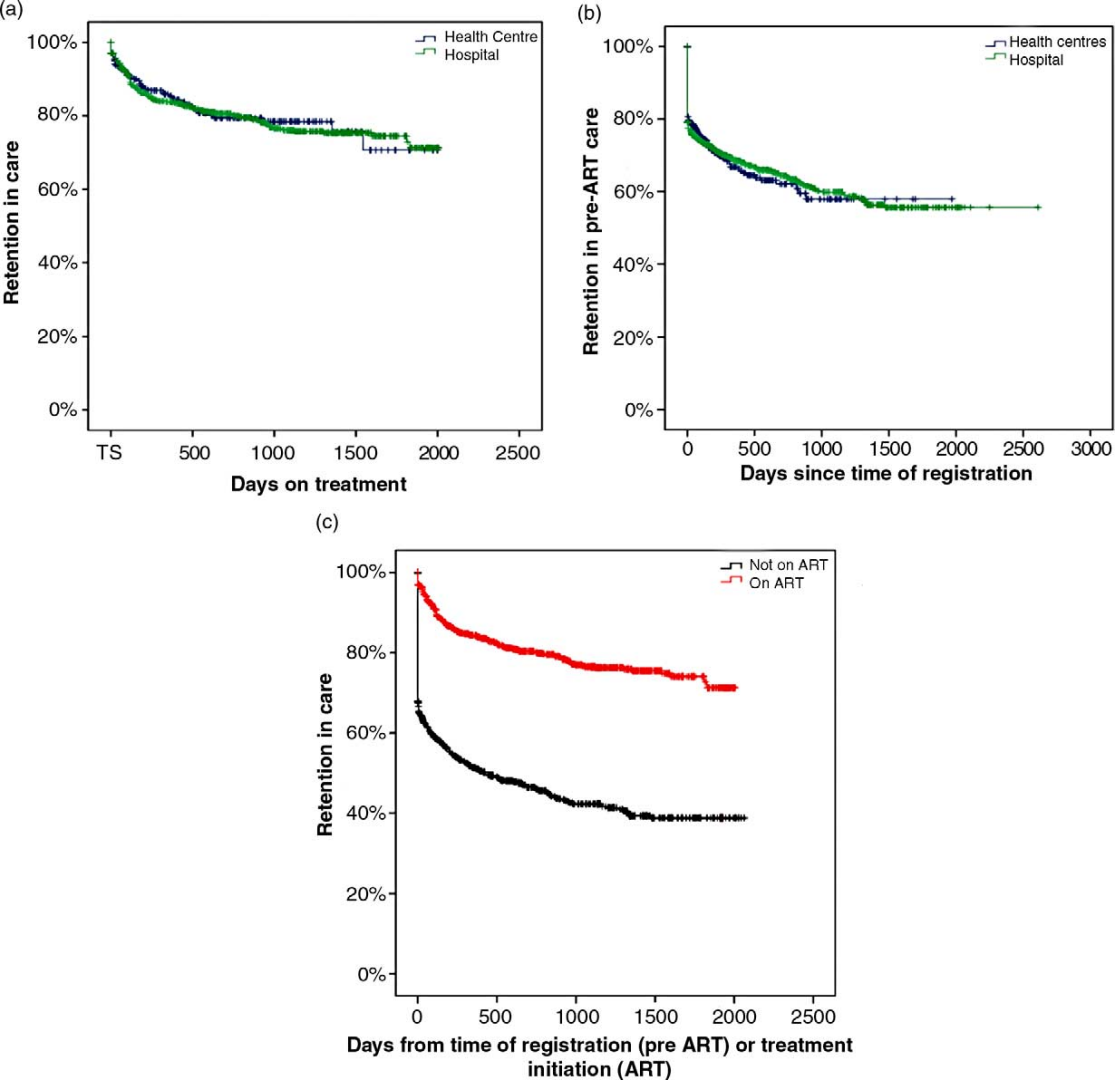
Retention in care [compared to adverse outcomes death and loss to follow-up (LTFU)] for different patient categories. Subjects are censored after their respective time of treatment and at time of registered transfer out. (a) Patients on antiretroviral therapy (ART patients), with regard to type of health facility. (b) Patients not having started ART (from enrolment to last follow-up or start of ART), with regard to type of health facility. (c) ART patients (from time of ART initiation) compared to pre-ART patients (from time of registration). Patients starting ART during follow-up are excluded from the pre-ART cohort.

The overall retention in care showed no significant difference between the two types of health facility (Fig. 1a, b), either for pre-ART or for ART patients (*p*=0.86 and *p*=0.85, respectively). Retention in care was significantly lower among pre-ART patients than among ART patients when all patients regardless of health facility were compared (*p*<0.001; Fig. 1c). The initial decrease among pre-ART patients represents subjects with only one clinic visit. A greater proportion of patients were transferred to other health facilities from the hospital clinic than from the health centres (16% vs. 9%).

### Growth after ART initiation

**Fig. 2:**
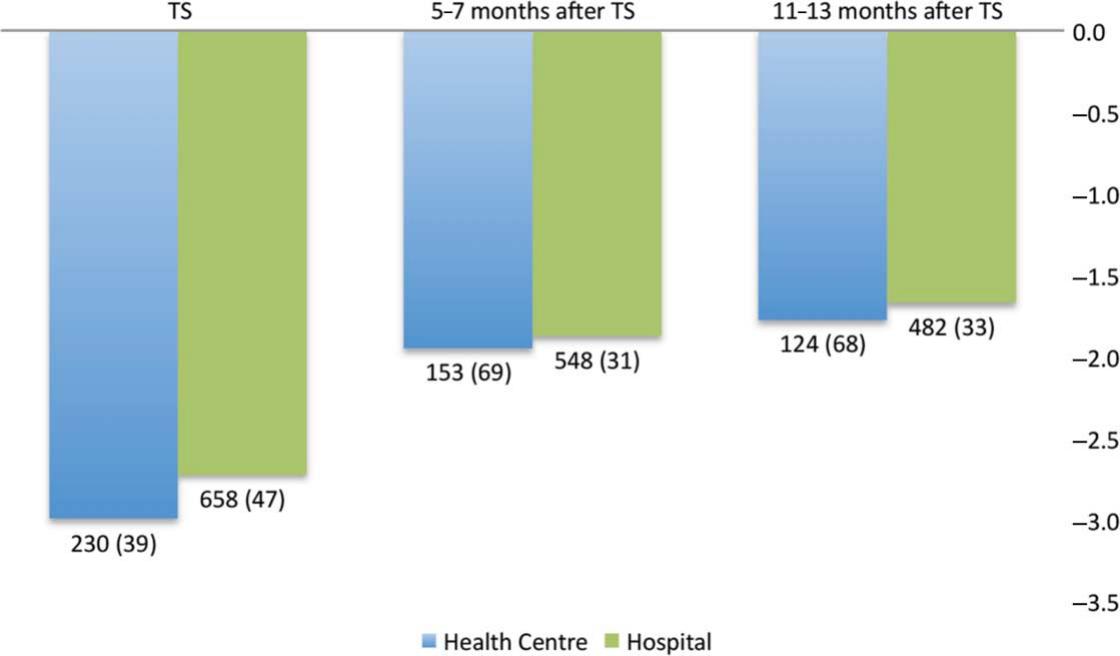
Median weight-for-age *z*-scores (WAZ) for patients on antiretroviral therapy (ART) during the first 13 months after ART initiation at three defined time points. A total of 31 outliers were excluded at baseline, four after 5–7 months, and one after 11–13 months. TS: *p*=0.098; 5–7 months: *p*=0.590; 11–13 months: *p*=0.466. The numbers of patients included (number of cases with missing WAZ data) are given under the columns. TS, treatment start (date at start of ART).

The proportions of children with malnutrition showed no significant difference between health centres and the hospital, and remained proportional during follow-up after initiation of ART, with increasing median WAZ scores during the first 13 months on ART (Fig. 2). Similar tendencies were observed for CD4 cell evolution, although follow-up results were unavailable for a majority of the children (data not shown).

### Risk factors for death and LTFU in patients on ART

In univariate analysis, the following factors were associated with mortality during the first 3 years on ART (Table 4): treatment in a health centre, age 0–1 years, WAZ scores below -4, and WHO clinical stage 4. In multivariate analysis, only age 0–1 years remained significant (HR 12.0; *p*<0.001).

**Table 4:** Risk factors for death in patients initiating antiretroviral therapy (ART) during follow-up

		Crude					Adjusted				
			
				95% CI for HR					95% CI for HR		
									
		No.	Hazard ratio	Lower	Upper	*p*	No.	Hazard ratio	Lower	Upper	*p*
Health facility	Hospital	578	1.00				463	1.00			
	Health center	222	2.12	1.03	4.36	**0.042**	159	1.94	0.68	5.50	0.213
	Missing	5					183				
Age groups	> 5 years	447	1.00				397	1.00			
	>1–5 years	223	1.21	0.44	3.32	0.718	185	1.60	0.51	5.05	0.421
	0–1 years	121	4.42	1.88	10.41	**0.001**	40	12.02	3.50	41.28	**<0.001**
	Missing	14					183				
Gender	Female	401	1.00				305	1.00			
	Male	399	2.02	0.94	4.31	0.070	317	1.89	0.70	5.10	0.209
	Missing	5					183				
Caretaker	Parents	531	1.00				414	1.00			
	Others	233	1.44	0.68	3.08	0.344	208	2.00	0.79	5.06	0.144
	Missing	41					183				
WAZ at TS	>–1	121	1.00				89	1.00			
	<–1 to > − 4	469	3.06	0.40	23.52	0.283	400	2.07	0.25	17.29	0.501
	< − 4	158	8.67	1.12	67.19	**0.039**	133	6.39	0.78	52.37	0.084
	Missing	57					183				
CD4 at TS	>350	252	1.00				231	1.00			
	<350	435	1.93	0.71	5.23	0.197	391	2.69	0.89	8.11	0.078
	Missing	118					183				
WHO clinical stage at TS	1, 2	341	1.00				270	1.00			
	3	379	1.24	0.53	2.90	0.623	307	1.21	0.42	3.44	0.723
	4	63	4.48	1.67	12.05	**0.003**	45	2.36	0.58	9.69	0.234

Patients lost to follow-up excluded from analysis. Significant p-values are marked with bold characters.

LTFU (Table 5) was associated with age 0–1 years and a WAZ score below -4 in univariate analysis. After adjustment, these factors remained significant; in addition, age 1–5 years and CD4 cell count below 350 cells/mm^3^ were associated with increased risk of LTFU.

**Table 5:** Risk factors for loss to follow-up (LTFU) in patients initiating ART

		Crude					Adjusted				
			
				95% CI for HR					95% CI for HR		
								
		No.	Hazard ratio	Lower	Upper	*p*	No	Hazard ratio	Lower	Upper	*p*
Health facility	Hospital	688	1.00				529	1.00			
	Health center	242	0.79	0.54	1.16	0.228	177	1.07	0.66	1.72	0.798
	Missing	14					238				
Age groups	5–15 years	501	1,00				444	1.00			
	1–5 years	263	1.43	0.98	2.08	0.066	215	1.61	1.03	2.53	**0.039**
	0–1 years	159	2.89	1.99	4.20	**<0.001**	47	3.31	1.65	6.64	**0.001**
	Missing	21					238				
Gender	Female	475	1.00				351	1.00			
	Male	454	0.92	0.68	1.26	0.618	355	0.94	0.64	1.38	0.747
	Missing	15					238				
Caretaker	Parents	621	1,00				469	1,00			
	Others	272	1.00	0.72	1.40	0.986	237	1.24	0.82	1.86	0.314
	Missing	51					238				
WAZ at TS	> − 1	135	1,00				98	1.00			
	< − 1 to- > − 4	530	1.21	0.70	2.12	495	448	1.39	0.70	2.76	0.352
	< − 4	198	2.48	1.39	4.40	**0.002**	160	2.77	1.35	5.65	**0.005**
	Missing	81					238				
CD4 at TS (cells/mm^3^)	>350	287	1.00				255	1.00			
	<350	501	1.18	0.81	1.72	0.393	451	1.85	1.15	2.98	**0.011**
	Missing	156					238				
WHO clinical stage at TS	1, 2	408	1,00				313	1.00			
	3	431	0.74	0.53	1.03	0.073	344	0.76	0.50	1.16	0.203
	4	69	1.02	0.56	1.83	0.960	49	0.80	0.35	1.80	0.584
	Missing	36					238				
TB treatment	Yes	120	1,00				102	1.00			
	No	810	1.28	0.78	2.09	0.325	604	1.07	0.60	1.92	0.824
	Missing	14					238				

Patients known to have died during follow-up excluded from analysis. TB, tuberculosis; TS, treatment start (date at start of ART); WAZ, weight-for-age *z*-score.

## Discussion

Rates of ART initiation and retention in care for HIV-infected Ethiopian children were similar in this population regardless of whether they received care in health centres or in a hospital clinic in the same uptake area. Although high proportions of subjects were lost to follow-up in both types of facilities, this outcome was significantly more common among hospital patients. These findings are in agreement with studies performed in other countries in sub-Saharan Africa (8, 24) and lend support to the feasibility of decentralising paediatric HIV care to public health centres.

Despite the introduction of paediatric HIV care at the health centre level, the majority of children in our uptake area were managed in the hospital clinic. Furthermore, patient characteristics differed with regard to type of health facility. Children in the hospital clinic had lower median CD4 cell counts, and higher proportions were infants. Since these factors were associated with mortality and LTFU in the total study population, the finding of comparable outcomes of care has to be interpreted with some caution. A similar pattern of differences in patient characteristics with regard to health facility (8), such as lower proportions of children below 24 months of age managed in primary health care facilities, was recently reported by Fayorsey et al. (8). Our observation of an increased risk of death in infants highlights the particular need of ART in this group of children, as well as the requirement for further research on the outcomes of infants treated in health centres.

Most patients not retained in care had unknown outcomes (LTFU), and rates of confirmed mortality were surprisingly low. Survival among patients not receiving care is known to be poor, and the fact that age below 1 year was identified as a risk factor both for death and LTFU in our population suggests that several children lost to follow-up may have died. Mortality was found to be the cause of LTFU among 40% of adult ART recipients in southern Ethiopia (25), and is likely to explain several cases of LTFU in our cohort. Consequently, reported mortality rates are probably underestimated. Children lost to follow-up were furthermore characterised by other baseline factors known to be associated with an elevated risk of death, such as CD4 cell counts below 350 cells/mm^3^ and higher degrees of wasting (14, 26, 27). The low retention rate in both kinds of health facilities illustrates a major problem in HIV care in resource-limited settings, which is common both among adults and children (28). This shows the need for improved tracing of patients who interrupt clinic attendance, and suggests that such programmes should be introduced in routine care for HIV-infected children.

Apart from increasing ART coverage for HIV-infected children, a better understanding of reasons for patient attrition is necessary in order to address underlying factors adequately. Certain facility characteristics associated with better retention in care have been reported from paediatric programmes in sub-Saharan Africa (29), for example, provision of nutritional support, early infant diagnosis, HIV support groups, and home-based care.

Our study is the first to compare outcomes in children receiving HIV care in health centres to those managed in a hospital clinic in Ethiopia. All public health facilities providing HIV care in the same uptake area were included, and a detailed review of patient characteristics in the respective health facilities was performed. We also analysed factors associated with adverse outcomes in this population, which allowed for a comparison of outcomes in light of differences in patient characteristics at the two types of facilities.

### Limitations

Our study was restricted to one geographical area of Ethiopia and the findings may not apply to other regions; however, we think that our uptake area is typical of public HIV care in low-income countries in sub-Saharan Africa. Furthermore, since no population-based data on HIV prevalence in different age groups exist for this area, it was impossible to assess the accuracy of the age distribution in our study population. This may be related both to insufficient antenatal HIV testing of pregnant women (1) as well as delayed infant HIV diagnosis before the introduction of PCR-based methods for this purpose in May 2008 (30).

As mentioned above, the high rate of LTFU in our population makes it difficult to definitely exclude the existence of differences in mortality between types of health facilities. Furthermore, the unknown proportion of deaths among patients lost to follow-up could have introduced bias in the analysis of risk factors for mortality. Due to the retrospective nature of this study, we were unable to investigate causes of LTFU in the population. Determination of immunological outcome was limited by the lack of follow-up CD4 cell data for a large proportion of participants. In order to assess and compare ART outcomes with greater accuracy, it would be necessary to examine rates of virological suppression, the most reliable method for the detection of treatment failure. Several studies have shown high rates of persistent HIV replication in children on ART and selection of drug resistance mutations (31, 32).

## Conclusions

No significant differences in rates of ART initiation and retention in care among children receiving HIV care in Ethiopia with regard to type of health facility were detected. However, patient characteristics were found to differ between the two types of health facilities, with subjects receiving care at the hospital clinic showing a higher prevalence of factors associated with subsequent death or LTFU. Our findings support continued integration of paediatric HIV care within the Ethiopian primary healthcare system but also call for more detailed studies on the outcome of ART and causes of LTFU in children, especially infants.
